# Viability of primary cultured podocytes is associated with extracellular high glucose-dependent autophagy downregulation

**DOI:** 10.1007/s11010-017-2949-5

**Published:** 2017-02-24

**Authors:** Irena Audzeyenka, Dorota Rogacka, Agnieszka Piwkowska, Stefan Angielski, Maciej Jankowski

**Affiliations:** 1 Department of Molecular and Cellular Nephrology, Mossakowski Medical Research Centre, Polish Academy of Sciences, Dębinki 7, 80-211 Gdansk, Poland; 2 Department of Clinical Chemistry, Medical University of Gdansk, Gdansk, Poland

**Keywords:** Autophagy, Podocyte, Insulin resistance, High glucose concentration, Diabetic nephropathy

## Abstract

Structural and functional impairment of podocytes plays an important role in the development of diabetic nephropathy, a chronic complication of diabetes mellitus and leading cause of renal failure requiring renal replacement therapy. Autophagy plays a crucial role in podocyte viability and function, and its activity is modulated by a variety of pathophysiological factors found in diabetic milieu. Here we show that downregulation of autophagy is critical for podocyte survival in hyperglycemic environment. Moreover, long-term exposure to high glucose leads to inhibition of autophagy as well as to the development of insulin resistance in podocytes. Furthermore, impairment of autophagy is involved in alteration of insulin-dependent glucose uptake in podocytes, suggesting a relationship between these two processes. Taken together, our findings suggest that downregulation of podocyte autophagy, observed after long-term exposure to high glucose, results from their suppressed sensitivity to insulin, and may therefore lead to diminished podocyte cell viability as well as their reduced number in glomerulus.

## Introduction

Podocytes play a key role in the regulation of glomerular permeability to albumin and maintain spatial structure of glomerulus by withstanding high intraglomerular hydrostatic pressure, mainly through a well-developed contractile apparatus formed by actin and myosin filament bundles [1]. These terminally differentiated epithelial cells are situated on the urinary side of glomerular capillaries and together with the glomerular basement membrane (GBM) and endothelial cells form renal filtration barrier. They are anchored to the GBM through their foot processes, and adjacent foot processes interdigitate, creating a narrow space (30–50 nm) with a complex set of membrane proteins, called glomerular slit diaphragm [2]. Within the renal filtration barrier podocytes are the cells that are uniquely insulin-sensitive and capable of increasing extracellular glucose uptake in response to insulin stimulation by facilitating glucose transporter translocation to cell membranes [3]. Altered morphology and reduced number of podocytes are the earliest pathological manifestations associated with diabetic nephropathy (DN), a leading cause of renal failure requiring renal replacement therapy in diabetic patients [4, 5]. Progressive development of insulin resistance observed at the early stage of type 2 diabetes causes an increase in blood glucose concentration (hyperglycemia) and compensatory rise of insulin levels [5]. Insulin resistance is characterized by a suppressed insulin-stimulated glucose uptake by the cells, resulting from the impairment of intracellular insulin signaling pathways and disability to increase the amount of glucose transporters (GLUTs) in the cell membrane [6]. Defective insulin responsiveness occurs as a consequence of genetic susceptibility and effects of extracellular agents [7], among which the excess of free fatty acids, chronic hyperglycemia and hyperinsulinemia are currently of particular research interest [8, 9].

Long-term hyperglycemia causes impairment of podocytes morphology and function, including foot processes effacement, hypertrophy, detachment from the GBM, and apoptosis, thus leading to the increased permeability of the filtration barrier to albumin and progressive decline of renal function [10]. Podocytes have very limited ability for cell division, therefore, their loss, although initially balanced by hypertrophy of the remaining podocytes, cannot be compensated by proliferation or efficient recruitment and differentiation of other glomerular cells [11, 12]. To endure adverse environmental changes, podocytes activate their intracellular defense mechanisms, among which autophagy is thought to be the most important one [13].

Autophagy serves as an adaptive mechanism aimed to remove improperly folded proteins and non-functional intracellular organelles, which might be produced during enhanced protein synthesis observed in cellular hypertrophy. Autophagic process involves a series of following steps including initiation, cargo recognition, autophagosome formation, autophagosome-lysosome fusion, digestion, and release of digested products into the cytoplasm. The process is initiated by Unc-51-like kinase-1/2 (ULK1/2) complex, under control of AMP-activated and the mammalian target of rapamycin (mTOR) kinases [14]. The membrane of the autophagosome is doubled in elongation process and its closure is regulated by Beclin1/PI3K class III complex, together with two conjugating systems involving LC3 protein processing and Atg12-Atg5-Atg16 complex [15]. Activity of autophagy is closely related to intracellular energy state, and all its signaling pathways are tightly regulated. Starvation, increased reactive oxygen species generation, or mechanical stress are the strong inducers of autophagy, aimed to maintain intracellular homeostasis in harsh conditions [16, 17].

Although the pathogenesis of DN remains to be fully elucidated, autophagy is currently considered as one of the key factors contributing to the disease [18–20]. Impaired autophagic activity in podocytes was observed in diabetic patients with proteinuria but not in those with no or minimal proteinuria [19]. It was also demonstrated on mice that podocyte-specific *Atg5* deletion and high-fat diet lead to albuminuria, activation of apoptosis in podocytes and their loss [19]. Moreover, inhibition of podocytes autophagy in mice (*Atg5* knockout) resulted in albuminuria by 2 months of age, renal segmental lesions with hyalinosis, and tuft-to-capsule adhesions by 4 months of age [21], indicating that proper podocyte autophagy is critical for normal kidney function. Additionally, recently published studies suggest that autophagy may be directly linked to the development of podocyte insulin resistance [22] and may constitute a protective mechanism against high glucose-dependent development of insulin resistance in these cells [23]; therefore, it is of interest to further investigate the significance of autophagy for viability and insulin responsiveness of primary rat podocytes exposed to high concentration of glucose or insulin.

## Materials and methods

### Reagents

Cell culture reagents were purchased from Sigma-Aldrich (St. Louis, MO, USA), with the exception of fetal bovine serum (FBS), which was purchased from Gibco, Invitrogen (Carlsbad, CA, USA). Rabbit polyclonal antibodies against LC3 and mouse monoclonal antibody against actin were obtained from Sigma-Aldrich. Alkaline phosphatase-conjugated secondary antibodies (goat anti-rabbit and donkey anti-mouse antibodies) were obtained from Santa Cruz Biotechnology (Santa Cruz, CA, USA). Small interfering RNA (siRNA) targeting Atg5 and its non-silencing control siRNA were obtained from OriGene (Rockville, MD, USA). Plasmid ptfLC3 was purchased from Addgene (plasmid 21,074). [1,2-^3^H]-deoxy-d-glucose was obtained from MP Biochemicals (Santa Ana, CA, USA). All other reagents were purchased from Sigma-Aldrich.

### Primary podocyte culture

All rats were maintained and studied in accordance with the protocol reviewed and approved by the local Bioethical Committee at the Medical University of Gdansk (No. 4/2012). Primary rat podocytes were obtained from Wistar female rats weighing 100–120 g as described previously [24]. Cell phenotypes were determined using podocyte-specific antibodies against Wilms’ tumor-1 protein (WT-1; Biotrend Koeln, Germany) and synaptopodin (Progen, Heidelberg, Germany). Depending on the experiment, podocytes were incubated in high glucose medium (HG; 30 mM d-glucose), osmotic control medium (Osm con; 11 mM d-glucose with 19 mM l-glucose), or standard glucose medium (SG; 11 mM d-glucose).

### Western blot analysis

Podocytes were washed with ice-cold PBS and homogenized in an ice-cold lysis buffer. The lysates were then centrifuged for 20 min at 15,000×*g*. Equal amounts of protein extracts (20 μg per well) were resolved by SDS-PAGE, transferred onto a PVDF membrane, and blocked with 3% fat-free milk in TBS (20 mM Tris-HCl, 140 mM NaCl). Next, the membrane was incubated overnight with primary antibodies against LC3 (1:500) and actin (1:3000). Following incubation with the appropriate secondary alkaline phosphatase-conjugated antibodies (1:5000), protein bands were detected using the colorimetric 5-bromo-4-chloro-3-indolyl phosphate/nitroblue tetrazolium (BCIP/NBT) system. Densitometric quantification of bands was performed with Quantity One software (Bio-Rad, Hercules, CA, USA).

### Nucleofection with plasmid DNA

Podocytes seeded on type I collagen-coated 24-well plates (Greiner Bio-One, Austria) at 70–80% confluency were transfected with an Amaxa AD1 Primary Cell 4D-Nucleofector-Y Kit (Lonza, Switzerland) and ptfLC3 plasmid (16 µg per well) using the FB-166 program on the 4D-Nucleofector System (Lonza). Immediately after nucleofection, cells were transferred into 1 ml of pre-warmed RPMI 1640 medium.

Cell were incubated in indicated media, and every 24-h 15–30, images were taken using an Olympus IX51 fluorescence microscope (Olympus, Japan). Total fluorescence signal was measured for autophagosomes and autolysosomes using CellSens Dimension software (Olympus, Japan). The results were presented as autolysosome to autophagosome fluorescence ratios.

### siRNA transfection

Podocytes were cultured in type I collagen-coated culture flasks in RPMI 1640 with 10% FBS and then transfected with siRNA Transfection Reagent (Santa Cruz Biotechnology) following the manufacturer’s instructions. Non-specific (scrambled) siRNA and Atg5 siRNA were diluted in Transfection Medium to a final concentration of 50 nM. Cells were cultured for 24 h and then incubated in SG, HG, or Osm con medium for 5 days. Parallel to monitoring autophagic vacuoles formation in Atg5 siRNA transfected viable podocytes, nucleofection using ptfLC3 was performed.

### MTT test

Podocytes were seeded at 3 × 10^4^/well on a type I collagen-coated 24-well plate, and cultured under control and experimental conditions. MTT (3-(4,5-dimethylthiazol-2-yl)-2,5-diphenyltetrazolium bromide) was added to each well to a final concentration of 0.2 mg/ml. Next cells were incubated at 37 °C for 4 h, medium was removed, and the intracellular formazan was solubilized in 300 μl DMSO. Absorbance was measured at 560 nm with a reference of 690 nm using a VICTOR^3^ Multilabel Counter 1420 (Perkin Elmer, Waltham, USA).

### [1,2-^3^H]-deoxy-d-glucose (2-DG) uptake measurements

Podocytes were cultured on a type I collagen-coated 24-well plate under control (SG) and experimental (SG supplemented with 300 nM insulin) conditions. On the day of experiment, cells were starved for 2 h in FBS- and glucose-free medium. Next [1,2-^3^H]-deoxy-d-glucose (2-DG) was added to a final concentration of 1 μCi/well and podocytes were incubated in 37 °C, 5% CO_2_ for 3 min. 400 μl of medium from each well was suspended in 2 ml of scintillator cocktail for extracellular radioactivity measurements by liquid scintillation counting (MicroBeta2^®^, Perkin Elmer, Waltham, USA). Remaining medium was removed, podocytes were washed three times with ice-cold PBS and lysed in 0.05 N NaOH for 1.5 h at room temperature. The lysates (400 μl/well) were transferred to scintillator cocktail (2 ml) and intracellular radioactivity was measured (MicroBeta2^®^, Perkin Elmer, Waltham, USA). The remaining lysates (100 μl) were used to determine the protein concentration using Bradford method [25].

### Statistics

All data are expressed as mean ± SEM. Quantitative comparisons were performed using ANOVA followed by the Student–Newman–Keuls multiple comparison test. A *P* value of <0.05 was considered significant.

## Results

### Autophagy is involved in extracellular high glucose concentration-dependent decrease in primary rat podocyte viability

**Fig. 1:**
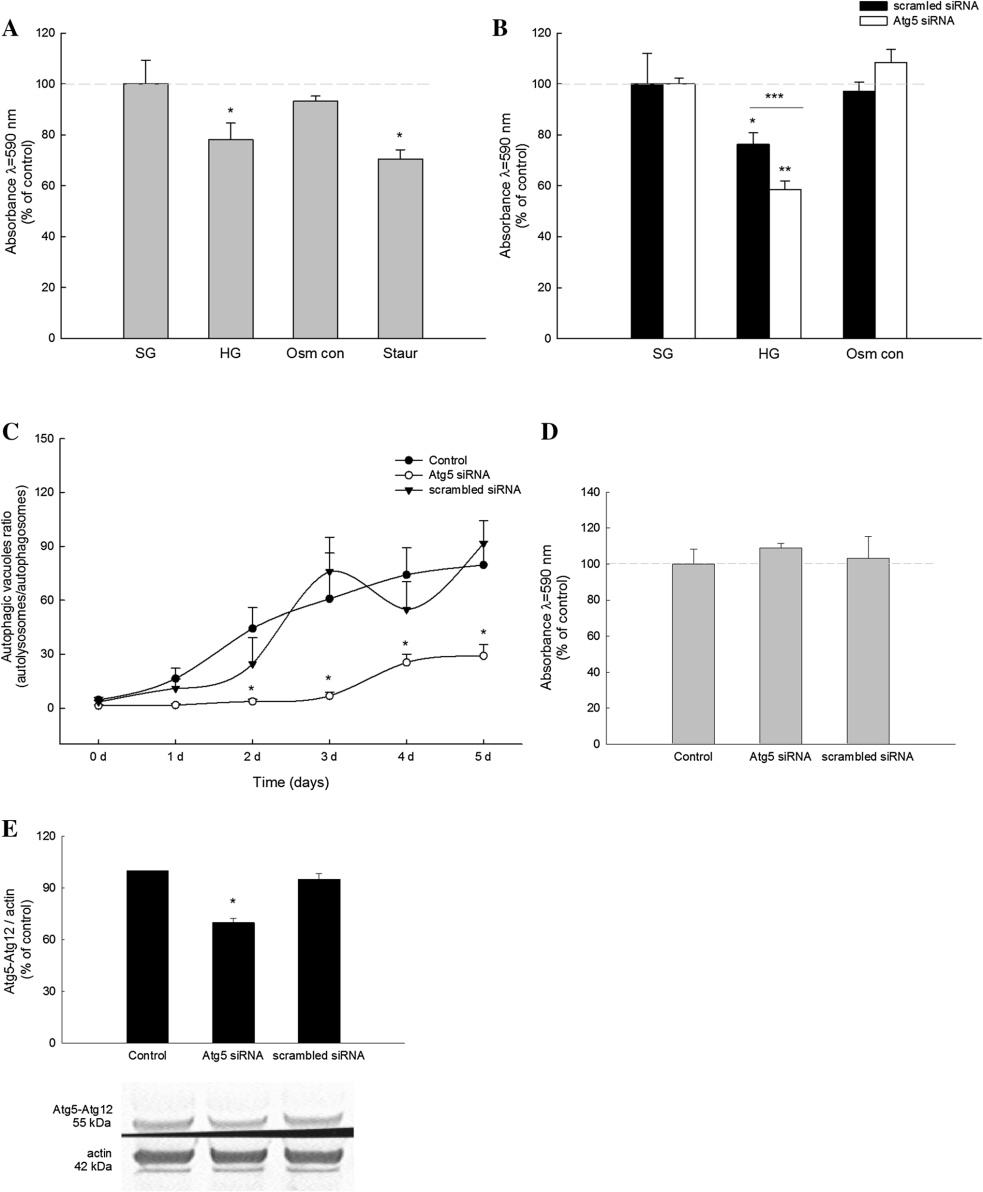
Effects of autophagy downregulation on primary rat podocyte viability (MTT test). Podocytes were cultured in high d-glucose (HG; 30 mM), standard d-glucose (SG; 11 mM), or in 11 mM d-glucose with 19 mM l-glucose (Osm con; osmotic control) for 5 days. **a** Viability of intact podocytes cultured in SG, HG and Osm con media for 5 days. The positive control was treated with staurosporine (Staur; 0.5 µM; 3 h). Values are means ± SEM (*n* = 3). *P < 0.05 versus SG. **b** Podocytes were transfected with Atg5 siRNA or scrambled siRNA and cultured in indicated media for 5 days. Values are means ± SEM (*n* = 3). *P < 0.05 versus SG scrambled siRNA, **P < 0.05 versus SG Atg5 siRNA, ***P < 0.05 scrambled siRNA versus Atg5 siRNA. **c** Intracellular autolysosomes to autophagosomes ratio after Atg5 siRNA transfection. Values are means ± SEM (*n* = 8–20). *P < 0.05 versus Control. **d** Atg5 siRNA and scrambled siRNA transfection effect on podocyte survival in standard medium (SG; 5 days). **e** Representative immunoblots for Atg5–Atg12 complexes in podocyte after transfection with siRNA for Atg5

In order to determine the viability of primary rat podocytes cultured in HG medium, MTT test was conducted. After 5 days of incubation in HG, we observed a reduction of metabolically active podocytes of about 22% in HG medium (30 mM d-glucose) when compared to SG medium (11 mM d-glucose) (*P* < 0.05), while in podocytes cultured for 5 days in SG and subsequent staurosporine stimulation (0.5 μM, 3 h; positive control), there was 30% decrease in cell viability (*P* < 0.05) (Fig. 1a).

Next, we investigated whether autophagy is involved in the reduced podocyte viability in HG. Incubation of Atg5-depleted podocytes in HG medium for 5 days led to the decline in cell viability of about 42% (*P* < 0.05) when compared to podocytes maintained in SG medium (Fig. 1b). Podocytes transfected with non-targeting siRNA (negative control) and cultured in HG for 5 days had abolished cell viability by 24% (*P* < 0.05) (Fig. 1b). To exclude the effect of elevated osmotic pressure in HG medium on podocytes, we assessed podocyte viability in non-transfected and transfected cells cultured for 5 days in high osmolarity (11 mM d-glucose with 19 mM l-glucose; Osm con) and SG medium (Fig. 1a, b). Autophagy downregulation in podocytes resulted in approximately 2,5-fold decrease in autolysosome/autophagosome ratio 5 days after the transfection (Fig. 1c).Transfection conditions did not affect podocytes viability (Fig. 1d). Podocyte transfection with Atg5 siRNA resulted in the decrease of Atg5–Atg12 complex formation by 30% (Fig. 1e).

### High glucose impairs activity of autophagy in primary rat podocytes

**Fig. 2:**
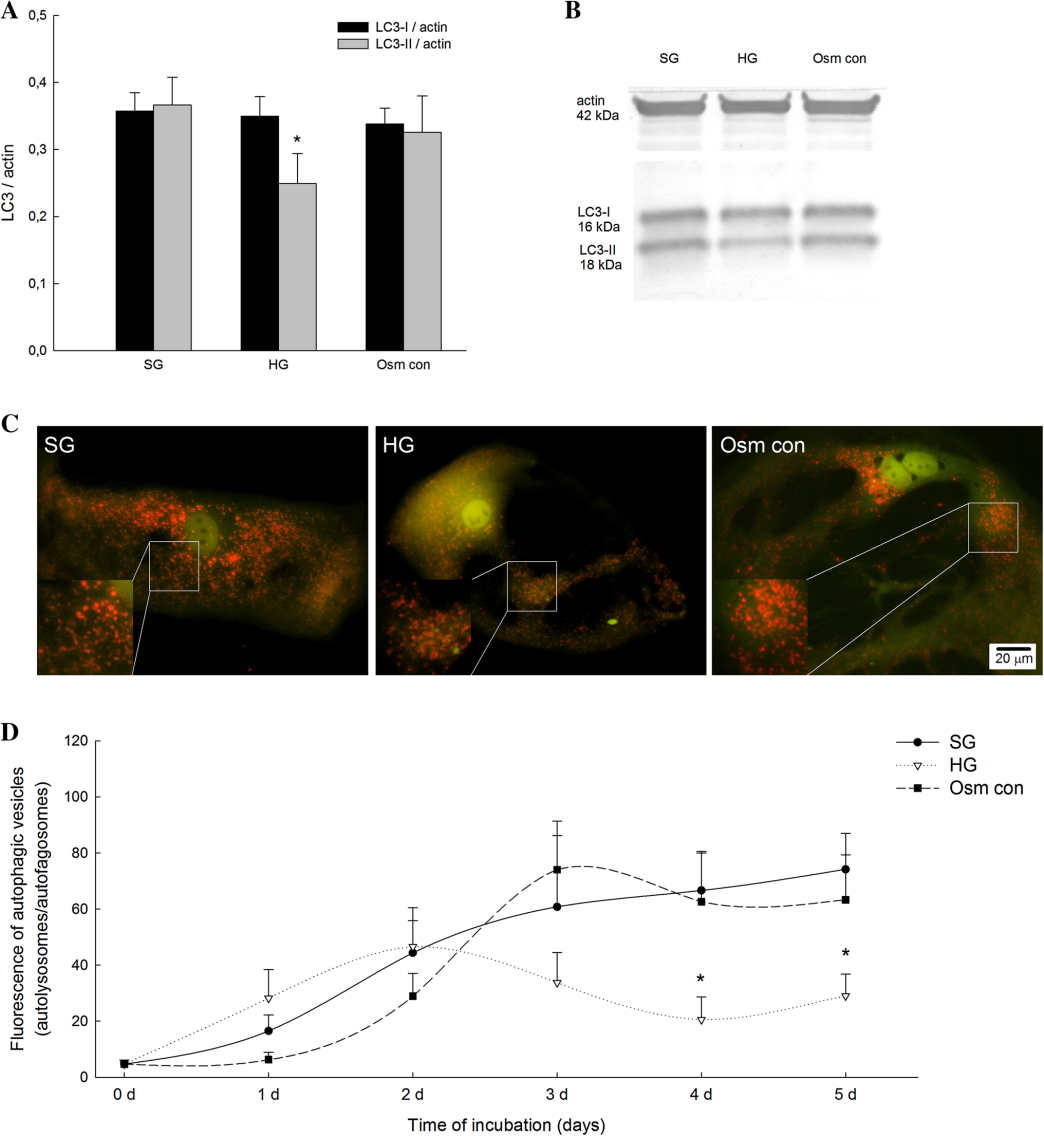
Effect of high glucose on LC3 expression in primary rat podocytes. Podocytes were cultured in high d-glucose (HG; 30 mM), standard d-glucose (SG; 11 mM), or 11 mM d-glucose with 19 mM l-glucose (Osm con; osmotic control) for 5 days. **a** Quantitative densitometric analysis was used to determine the ratios of LC3-I to actin and LC3-II to actin. Values are means ± SEM (*n* = 12). *P < 0.05 versus SG LC3-II/actin. **b** Representative immunoblot. **c** Representative images of intracellular localization of GFP-RFP-LC3 protein in podocytes after nucleofection and incubation in the indicated media for 5 days. **d** Autophagy flux analyses in podocytes after GFP-RFP-LC3 nucleofection. Total fluorescence for *red* (RFP; autolysosomes) and *yellow* (GFP and RFP; autophagosomes) emission signals was determined in each image. Values are means ± SEM (*n* = 10–20 images for each cell culture). (Color figure online)

Autophagy flux was analyzed in primary rat podocytes transfected by nucleofection with ptfLC3 plasmid expressing RFP-GFP-LC3 construct, which enables tracing autophagosome and autolysosome formation in viable cells within the course of time. After 5 days of podocyte incubation in HG medium, we observed significant decrease in LC3-II protein expression of about 32% when compared to SG medium (0.37 ± 0.04 vs. 0.25 ± 0.05, *P* < 0.05) (Fig. 2a, b). The LC3-I level was unchanged in podocytes cultured in both SG and HG (Fig. 2a, b). Reduced number of autolysosomes (red dots) toward autophagosomes (yellow/green dots) was observed in the fluorescence images of podocytes cultured for 5 days in HG medium (Fig. 2c), what was confirmed in the autophagy flux analyses (Fig. 2d).

### Downregulation of autophagy alleviates podocyte responsiveness to insulin

**Fig. 3:**
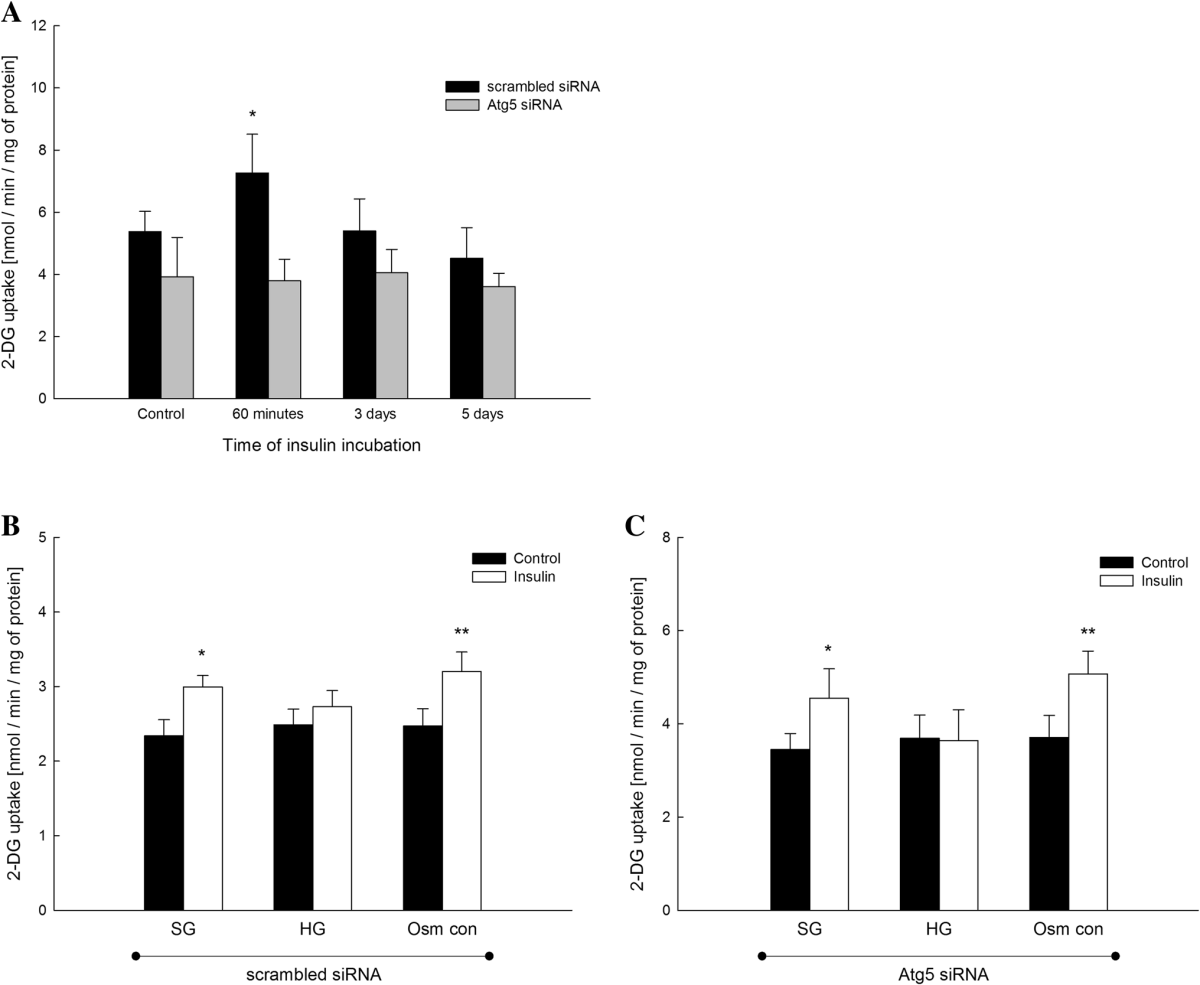
Involvement of autophagy in the development of insulin resistance in primary rat podocytes. **a** Measurements of [1,2-^3^H]-deoxy-d-glucose (2-DG) intracellular transport in podocytes transfected with Atg5 siRNA or scrambled siRNA and treated with insulin (300 nM) for the indicated times. Values are means ± SEM (*n* = 4–8). *P < 0.05 versus Control scrambled siRNA. **b** Measurements of insulin-dependent 2-DG uptake in podocytes transfected with scrambled siRNA and incubated in standard d-glucose (SG; 11 mM), high d-glucose (HG; 30 mM), or with 11 mM d-glucose with 19 mM l-glucose (Osm con; osmotic control) for 5 days. Insulin stimulation (300 nM) was performed during 3 min. Values are means ± SEM (*n* = 5). *P < 0.05 versus SG Control, **P < 0.05 versus Osm con Control. **c** Measurements of insulin-dependent 2-DG uptake in podocytes transfected with Atg5 siRNA and incubated in SG, HG, or Osm con for 5 days. Insulin stimulation (300 nM) was performed for 3 min. Values are means ± SEM (*n* = 7). *P < 0.05 versus SG Control, **P < 0.05 versus Osm con Control

In order to determine if autophagy plays an important role in podocyte sensitivity to insulin, we transfected podocytes with Atg5 siRNA or non-specific control siRNA and analyzed time-dependent effects of insulin (300 nM) on intracellular [1,2-^3^H]-deoxy-d-glucose (2-DG) uptake. The uptake of 2-DG in podocytes transfected with control siRNA was increased by 35% after 60 min of insulin stimulation (5.38 ± 0.64 nmol/min/mg protein vs. 7.27 ± 1.23 nmol/min/mg protein, *P* < 0.05) (Fig. 3a), whereas 3 as well as 5 days of stimulation with insulin abolished this effect (control: 5.38 ± 0.64 nmol/min/mg protein; 3-days insulin incubation: 5.4 ± 1.03 nmol/min/mg; 5-days insulin incubation: 4.52 ± 0.98 nmol/min/mg protein) (Fig. 3a). However, in Atg5-depleted podocytes, we did not observe any effect of insulin on intracellular 2-DG transport at any of the time points (Fig. 3a).

To determine the involvement of Atg5 in sensitivity of primary rat podocytes to insulin, we analyzed insulin-dependent uptake of 2-DG into the podocytes transfected with Atg5 siRNA or non-targeting siRNA after subsequent incubation in SG, HG, and Osm con media for 5 days. In podocytes transfected with non-specific siRNA and cultured in SG or Osm con, short stimulation with insulin (300 nM, 3 min) resulted in significantly increased 2-DG transport into the cells by about 28 and 29%, respectively (2.34 ± 0.22 nmol/min/mg protein vs. 2.99 ± 0.15 nmol/min/mg protein (SG) and 2.47 ± 0.23 nmol/min/mg protein vs. 3.2 ± 0.26 nmol/min/mg protein (Osm con), *P* < 0.05), while there was no effect of insulin on 2-DG uptake into podocytes cultured for 5 days in HG medium (Fig. 3b). Similar effect was observed in podocytes with downregulated autophagy, where stimulation with insulin (300 nM, 3 min) caused significant increase in 2-DG transport after incubation in SG medium (Δ32%, *P* < 0.05) or Osm con medium (Δ37%, *P* < 0.05) for 5 days, whereas there was no effect of insulin on 2-DG uptake in podocytes cultures in HG medium (Fig. 3c).

## Discussion

Autophagy constitutes one of the most important defense mechanisms involved in maintaining podocytes intracellular homeostasis by removing misfolded proteins and non-functional organelles [20, 26]. Basal activity of podocytes autophagy, the highest among all the kidney cells [13], is associated with high metabolic activity of these non-dividing cells to sustain permanent filtration of large volume of serum (600 ml/min in human) and low-molecular-weight proteins, including albumin (up to several g/day), and result in the necessity of constant intracellular proteomic turnover. DN is characterized by the impairment of renal filtration mainly due to alterations in podocyte morphology and function, therefore multiple studies have been trying to elucidate the potential effects of diabetic milieu on podocyte physiology. Previously published reports do not provide unequivocal answer whether autophagy is activated or inhibited in high glucose environment (HG). It has been shown that number of autophagosomes in podocytes in diabetic rats is increased. Moreover, increased LC3 and beclin1 expression was shown in immortalized mice podocytes cultured in HG [27]. In contrary, it has also been shown that both markers of autophagy as well as number of autophagic vacuoles were decreased in different models of experimental diabetes [28]. Recent study has shown a decrease in the LC3-II expression and in the number of autophagic vesicles in podocytes of diabetic patients and rodents. Moreover, decreased podocyte viability has been observed in HG [19]. In our work, we have shown that long-term exposure of primary rat podocytes to high glucose indeed promotes podocyte death, and attenuation of autophagy further deteriorates this process. Experimental conditions, which were used to inhibit podocyte autophagy, did not affect cell viability in control environment, therefore indicating that podocytes are capable to compensate mild disturbances in autophagy activity, however, not under long-term exposure to unfavorable milieu. Additionally, we observed a reduced expression of LC3-II and a decreased number of intracellular autophagic vacuoles in podocytes cultured for 5 days in HG, when compared with both standard medium and osmotic control. Thus, there was no effect of the osmotic pressure on the autophagy activity in cells cultured in HG. In our previous work, we have shown that podocytes cultured for 5 days in HG failed to increase d-glucose uptake in response to insulin treatment [29]. Moreover, variety of cell types has been reported to develop insulin resistance following the long-term insulin treatment [9, 30, 31]. In the current work, we have shown for the first time that mild inhibition of autophagy activity is sufficient to impair responsiveness of podocytes to insulin that was observed in cells transfected with Atg5 siRNA. 2-DG uptake was unchanged after 60 min of insulin treatment, whereas in control podocytes, intracellular 2-DG transport was increased by 35%. After long-term incubation with insulin (3 and 5 days), there was no effect of insulin on 2-DG uptake in podocytes transfected with Atg5 or scrambled siRNA, what may indicate the development of insulin resistance in podocytes. These results are in agreement with recently published work of Xu et al., where the authors observed decreased autophagy in immortalized human podocytes after knocking down the insulin receptor, suggesting that proper insulin signaling is critical for autophagy in podocytes [22]. We have also shown direct influence of insulin on the expression of markers of autophagy in podocytes, as well as on their permeability to albumin [24]. In our present study, we have also investigated if downregulation of autophagy has any effect on the 2-DG uptake in podocytes cultured in HG. Here, in both transfected with Atg5 or scrambled siRNA cell cultures, we observed abolished effect of HG on insulin-dependent intracellular 2-DG transport, indicating that preceding downregulation of Atg5 signaling, which may impair autophagy activity, does not influence insulin resistance development after long-term HG treatment. However, it should be noted that high percentage of podocytes dying in HG may also indicate a progressive deterioration of remaining cell function and viability, which might involve a dysregulation of various intracellular pathways, including insulin signaling pathway. Therefore, this aspect can be a promising field for further investigation.

Taking under account all studies conducted so far, it seems possible, that downregulation of autophagy, that is observed in podocytes exposed to hyperglycemic environment, can represent the effect of reduced sensitivity of podocytes to insulin elicited by long-term exposure to HG, and may also contribute to the decreased viability of these cells.
